# Caregiver Expectations of Interfacing With Voice Assistants to Support Complex Home Care: Mixed Methods Study

**DOI:** 10.2196/37688

**Published:** 2022-06-30

**Authors:** Ryan Tennant, Sana Allana, Kate Mercer, Catherine M Burns

**Affiliations:** 1 Department of Systems Design Engineering Faculty of Engineering University of Waterloo Waterloo, ON Canada; 2 Library University of Waterloo Waterloo, ON Canada

**Keywords:** caregivers, children, communication, digital, home care, information management, interaction, older adult, technology acceptance, voice assistant

## Abstract

**Background:**

Providing care in home environments is complex, and often the pressure is on caregivers to document information and ensure care continuity. Digital information management and communication technologies may support care coordination among caregivers. However, they have yet to be adopted in this context, partly because of issues with supporting long-term disease progression and caregiver anxiety. Voice assistant (VA) technology is a promising method for interfacing with digital health information that may aid in multiple aspects of being a caregiver, thereby influencing adoption. Understanding the expectations for VAs to support caregivers is fundamental to inform the practical development of this technology.

**Objective:**

This study explored caregivers’ perspectives on using VA technology to support caregiving and inform the design of future digital technologies in complex home care.

**Methods:**

This study was part of a larger study of caregivers across North America on the design of digital health technologies to support health communication and information management in complex home care. Caregivers included parents, guardians, and hired caregivers such as personal support workers and home care nurses. Video interviews were conducted with caregivers to capture their mental models on the potential application of VAs in complex home care and were theoretically analyzed using the technology acceptance model. Interviews were followed up with Likert-scale questions exploring perspectives on other VA applications beyond participants’ initial perceptions.

**Results:**

Data were collected from 22 caregivers, and 3 themes were identified: caregivers’ *perceived usefulness* of VAs in supporting documentation, care coordination, and person-centered care; caregivers’ *perceived ease of use* in navigating information efficiently (they also had usability concerns with this interaction method); and caregivers’ concerns, excitement, expected costs, and previous experience with VAs that influenced their *attitudes toward use*. From the Likert-scale questions, most participants (21/22, 95%) agreed that VAs should support prompted information recording and retrieval, and all participants (22/22, 100%) agreed that they should provide reminders. They also agreed that VAs should support them in an emergency (18/22, 82%)—but only for calling emergency services—and guide caregivers through tasks (21/22, 95%). However, participants were less agreeable on VAs expressing a personality (14/22, 64%)—concerned they would manipulate caregivers’ perceptions—and listening ambiently to remind caregivers about their documentation (16/22, 73%). They were much less agreeable about VAs providing unprompted assistance on caregiving tasks (9/22, 41%).

**Conclusions:**

The interviews and Likert-scale results point toward the potential for VAs to support family caregivers and hired caregivers by easing their information management and health communication at home. However, beyond information interaction, the potential impact of VA personality traits on caregivers’ perceptions of the care situation and the passive collection of audio data to improve user experience through context-specific interactions are critical design considerations that should be further examined.

## Introduction

### Background

Although engaging in natural spoken conversation is the most common way of communicating information, humans are increasingly interacting with information through computers. The Turing test is often used to determine whether an exchange with a computer can be distinguished from that with a human, measuring the humanness of the interaction [1]. Significant research has been working toward imitating natural language conversations. However, this area has not yet been fully realized as a prominent means of human-computer interaction [2-4]. With advancements in natural language understanding and speech processing, the adoption of voice assistant (VA) technology such as Apple’s Siri, Amazon’s Alexa, Microsoft’s Cortana, and Google’s Assistant is increasing. This rise in adoption is primarily due to the ability of VAs to reduce barriers to accessing information, social attributes influencing the development of trust, and significant advancements in the technology [4-6]. Although VAs are commonly used to support everyday activities such as playing music, checking the weather, and listening to the news, emerging research explores potential health care applications [7-10].

Interacting with digital health technologies through a VA may provide a more natural, intuitive, and efficient way to engage with health information in complex home care by family members and their caregiving teams [11-13]. VAs may positively affect caregiver burnout by better supporting care coordination [14,15], where vocal recordings of health events and documentation could relieve a caregiver’s documentation burdens [13]. For children with special health care needs, VAs may support autonomy to self-manage health information as they transition to adulthood [12]. At the same time, for older adults, VAs have demonstrated improvements in independent living and health maintenance [16-19].

With the increase in individuals providing home care, especially during the COVID-19 pandemic, there is significant potential for VAs to support caregivers in this context [10,13]. In 2020, approximately 1 in 5 Americans were providing home care, with an increasing number of family caregivers reporting difficulties coordinating care with other caregivers [20]. Despite the COVID-19 pandemic bringing telehealth to the forefront and the desire for integrated information technologies, there remains a lack of standardized, easy-to-use systems to support communication and coordination among caregivers in complex home care [21]. VAs may provide an interaction method that is more suitable for this health care delivery context given the atmosphere of a home environment. However, it is unclear how caregivers would expect to interact with health information using VAs, which is critical for informing their design.

### Advancements in Digital Technologies for Home Care

Collaboration among caregivers is critical to ensure safety and quality care in someone’s home, especially when living with complex medical conditions and health service needs [13,22-25]. Mobile apps are a promising solution to support caregiver collaboration in the home, where computer use has become ubiquitous as a technology to enhance communication and information sharing. Nursing agencies currently use mobile apps to share care updates among their teams. However, these apps are often limited to the nursing team without including the family caregiver, who ultimately develops their own information management and communication methods in the home [26]. For family caregivers supporting older adults with dementia, the design of mobile apps to meet their information and communication needs has been shown to improve caregiving confidence, depression and self-efficacy, and interaction between caregivers and health care professionals [27-29]. Mobile apps have also been shown to ease information access on the part of caregivers to scientific knowledge about their children’s complex medical conditions [30].

There is increasingly more research on mobile app design, including a user-centered approach through qualitative data analysis where participants’ insights and expressed needs are used to direct feature and functionality development [27,31]. These short-term deployment studies highlight the impact of the novelty factor on the interest in integrating a mobile app on the part of caregivers. However, common challenges from research on mobile app use in complex home care centers on the apps’ inability to provide long-term flexibility as health conditions change or to support caregiver anxiety related to potential disease progression [30,31].

VAs may provide a way to support long-term health information management through their mode of interaction along with conversational aspects of interaction that could provide social support. Possible areas of benefit of VAs have been identified for hands-free documentation and data retrieval from electronic health records by health care professionals and for intelligent multimodal assistance by supporting telehealth use or detecting respiratory conditions [10,32]. In the context of home care, much of the current literature focuses on how older adults could interact with VAs, including medication timing and dosage reminders or encouraging physical activity [9,19]. With the rising age of our population, approximately one-third of dementia caregivers are older adults (aged >65 years) [8]. In general, older adults perceive the potential of VAs to improve their access to health information and their experiences in searching for information [33,34]. They also have concerns regarding privacy, financial burdens, and the accuracy of the information supplied. The perception of using VAs for a conversational interaction has resulted in mixed findings [33-35].

Other applications of VAs for home care have examined their use by caregivers to support older adults in aging in place and finding information, as well as for entertainment [8,35]. VAs have been designed to help caregivers manage the diet of someone diagnosed with dementia and provide guidance and personalized recommendations on nutrition, cooking, and eating behaviors [8]. Caregivers have also expressed their desire to use VAs to check in on medication events [35]. However, some of these developed systems have not been evaluated in a home care setting. Systems that have been evaluated in home care settings still experienced usability issues when integrating them into practice as the caregivers relied on paper-based tools to meet information management requirements [8,35]. There is an opportunity to use a user-centered approach to uncover aspects of VA design that should be considered to better meet the integration needs of caregivers through mixed research methods.

For caregivers of children with special health care needs, there is limited research on the potential of VAs to support health care tasks in the home. However, a spectrum of contexts for VAs has been proposed, ranging from general information retrieval to potentially prescribing therapy, medications, or other treatments [13]. VAs could also provide more autonomy to the children as they become teenagers and take more control over their health [13]. Preliminary work has shown positive attitudes toward VAs built into a medical diary app [7]. However, critical considerations and limitations preventing integration remain. For example, current limitations include access to raw health care data from mainstream vendors, Health Insurance Portability and Accountability Act compliance, the relative market demand, caregivers’ social and economic status, language support, and translating current services to permit voice interaction [13].

With the potential of VAs around home care support, it is critical to better understand stakeholders’ perspectives in a way that informs safe, accessible, and effective system design [10]. Few studies have explored caregivers’ attitudes toward designing intelligent home-based technologies such as VAs and how they may benefit caregiving [36]. With the rise in complex home care, there is an identified need to understand the human factors influencing caregivers’ perception of the usefulness and ease of use of VAs, and their attitudes toward using VAs to support technology adoption [36].

### Study Objective

The objective of this study was to explore caregivers’ initial perspectives on VA functionality that may influence future development and ultimately adoption of this technology using the technology acceptance model (TAM) and quantitative Likert scales. Given the collaborative nature of home care, this study included family and hired caregivers’ perceptions of using VAs to interface with health information and support care coordination.

## Methods

### Research Design

This research is part of a larger study to identify caregivers’ perspectives on information management and communication in complex home care and the design and use of VAs to support caregivers of children with special health care needs and older adults [26]. Taking a pragmatic stance, the researchers specifically recognized that a constructivist approach to the truth must acknowledge the continuum of experiences and perspectives related to experiences, illuminating the drivers of behavior [37,38]. This paper focuses on semistructured interviews and Likert-scale question results for caregiver participants’ expectations of VA functionality. The analysis was guided using the framework analysis method, which was chosen as it uses a systematic and intentionally flexible approach to analyzing multidisciplinary health and engineering data [39].

### Ethics Approval

The University of Waterloo Office of Research Ethics approved this study (Office of Research Ethics 42179). All participants were interviewed via Microsoft Teams because of the COVID-19 protection measures. Informed consent was obtained verbally, and the participants received a thank-you letter for taking part in this study.

### Participants and Data Collection

The research team recruited participants through home health care and caregiving agencies, social media groups, and snowball sampling. The recruitment objective was to engage participants with diverse backgrounds, ages, caregiving experiences, and experiences with VA technology in their homes. Eligible participants were either family caregivers or hired caregivers of adults or children who required complex care services in their homes in North America. In this study, complex care was defined as individuals with any combination of the following: complex chronic conditions, mental health issues, medication-related problems, and social vulnerability. A family caregiver was anyone who provided or coordinated care for a family member in their home: a parent, grandparent, guardian, spouse, child, or sibling. A hired caregiver was anyone who was paid to provide care in someone’s home: a personal support worker (PSW) or a nurse that provides home care services. Participants were not required to have previous experience with VAs. Before starting the interview, the researchers explained to the participants that VAs are a technology that allows humans to interact with information on a computer system through voice and audio—the participants did not explicitly interact with a VA in this part of the research study.

In total, 2 researchers (RT and KM) conducted the interviews. First, the caregivers were asked to describe their current experiences with VAs in their daily activities. Second, the caregivers were asked to describe their initial beliefs and expectations regarding VAs to support their caregiving work domain. At the end of the interview, the participants were asked 12 Likert-scale questions about their expectations of VAs in a home care context. The participants were asked to verbally respond to each question on a 7-point scale ranging from *strongly disagree* to *strongly agree*. Microsoft Teams was used to record the interviews, and only the audio recordings were stored for transcription.

### Data Analysis

The interview data on the participants’ expectations of VAs in complex home care were analyzed using a theoretical thematic process [40]. The TAM—a sociotechnical framework that posits that the adoption of a technology is driven by its capabilities and the effort required to use the technology—informed the identification of concepts and their interconnections for caregiver behaviors toward VAs in home care [41], an application context that has yet to be explored using the TAM. Although the TAM has been built upon since it was originally proposed, the fundamental framework has been successfully applied in information and communication technology in health care [42,43]. In this study, the data analysis focused on identifying the theoretical factors influencing potential usefulness, ease of use, and attitudes toward implementing VAs in home care. The usability attributes by Nielsen [44] guided the classification of the external variables influencing these 3 factors of the TAM.

First, the interviews were transcribed verbatim, and all names and identifiers were made anonymous. The research team listened to the interview recordings and read through the transcripts to familiarize themselves with the data. Core team members discussed each interview, thematically coded the data, and regularly met to discuss emerging concepts and themes. The final code list was organized into concepts and themes and presented to the entire research team for discussion and refinement. The Likert-scale results were triangulated with the participants’ qualitative responses and represented graphically while also contributing to subtheme development. These quantitative results were further broken down to visualize the expectations of participants who reported different levels of experience with VAs in their lives.

## Results

### Participant Demographics

There were 22 caregivers who participated in this study (Table 1). The participants were grouped by caregiver type, including family caregivers of older adults, hired caregivers of older adults, and family caregivers of children with special health care needs. The participants were recruited from various regions across Canada and the United States. The youngest participant in this study was aged 24 years, and the oldest was aged 83 years. Most of the participants identified as female (20/22, 91%), whereas 9% (2/22) identified as male. The participants’ caregiving experience ranged from 4 months to 13 years. More participants reported having minimal experience with VAs (12/22, 55%) than those who did have experience with VAs (10/22, 45%). Having minimal experience was defined as understanding the concept and existence of VA technology but having little to no experience interacting with one. Being experienced was defined as owning and interacting regularly with a VA smart speaker or a VA on a mobile device.

**Table 1:** Participant demographics and caregiving characteristics (N=22).

Characteristics		Family caregivers of children with special health care needs (n=7), n (%)	Family caregivers of older adults (n=9), n (%)	Hired caregivers of older adults (n=6), n (%)
**Age (years)**				
	18 to 24	0 (0)	1 (11)	0 (0)
	25 to 34	2 (29)	1 (11)	1 (17)
	35 to 44	5 (71)	0 (0)	2 (33)
	45 to 54	0 (0)	0 (0)	1 (17)
	55 to 64	0 (0)	1 (11)	1 (17)
	65 to 74	0 (0)	2 (22)	1 (17)
	75 to 84	0 (0)	4 (44)	0 (0)
**Gender**				
	Female	7 (100)	8 (89)	5 (83)
	Male	0 (0)	1 (11)	1 (17)
**Caregiving experience (years)**				
	0 to 5	1 (14)	6 (67)	4 (67)
	6 to 10	3 (43)	2 (22)	1 (17)
	11 to 15	2 (29)	1 (11)	1 (17)
	16 to 20	1 (14)	0 (0)	0 (0)
**Voice assistant experience**				
	Minimal experience	4 (57)	5 (56)	2 (33)
	Experienced	3 (43)	3 (33)	4 (67)
	Unknown	0 (0)	1 (11)	0 (0)

### Themes

#### Overview

**Figure 1:**
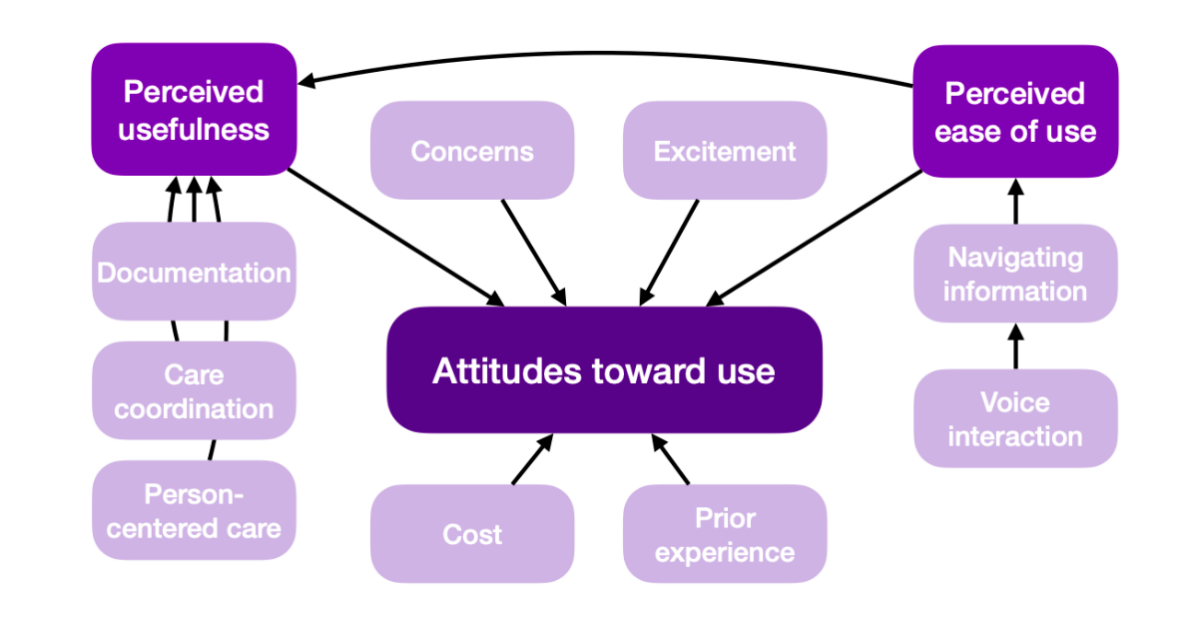
Caregiving factors influencing usefulness, ease of use, and attitudes toward using a voice assistant in complex home care.

The TAM was used to organize the qualitative findings of the participants’ initial beliefs and expectations regarding VA functionality in complex home care based on their current knowledge and experiences. There were 25 identified concepts that were originally organized into 8 subthemes. Structured within the TAM framework (Figure 1), the similarities among participant groups supported the merging of the subthemes into 3 themes (Table 2). An additional underlying subtheme of *prior experience* was identified after further analysis of the complete data set to comprise a total of 9 subthemes.

**Table 2:** Participants’ expectations of voice assistants in complex home care (N=22).

Theme, subtheme, and concept			A^a^	B^b^	C^c^
**Perceived usefulness**					
	**Documentation**				
		Organizing information	✓		
		Recording and retrieving information	✓	✓	✓
	**Care coordination**				
		Teaching caregivers through instructions	✓	✓	✓
		Reminding caregivers	✓	✓	✓
		Leaving messages for caregivers		✓	✓
		Calling others	✓	✓	✓
		Supporting physical tasks	✓	✓	✓
	**Person-centered care**				
		Providing autonomy for care	✓	✓	✓
		Supporting mild cognitive impairment		✓	✓
		Supporting medication management	✓	✓	✓
**Perceived ease of use**					
	**Navigating information efficiently**				
		Interacting by voice	✓	✓	
		Supporting aftercare	✓		
		Information retrieval		✓	
	**Usability concerns**				
		Being misunderstood or unheard	✓	✓	
		Engagement by the caregiver team			✓
		Challenging interfacing with computers		✓	✓
		Negative influence on physical activity			✓
**Attitudes toward use**					
	**Implementation concerns**				
		Standard for documentation			✓
		Medication management	✓		
		Privacy of information	✓	✓	✓
	**Excitement**				
		Learning new technology	✓	✓	
		Appreciation for voice-based technology		✓	
		Excitement about home care technology			✓
	**Cost**				
		Environmental benefits			✓
		Financial cost of the system	✓		

^a^Family caregivers of children with special health care needs.

^b^Family caregivers of older adults.

^c^Hired caregivers of older adults.

#### Perceived Usefulness

Despite their varied experiences with VAs, family caregiver and hired caregiver participants discussed VA design features that would provide utility to their home care situations, which were organized into three subthemes: (1) documentation, (2) care coordination, and (3) person-centered care. First, the participants believed that VAs would be helpful as a digital tool for managing their documentation by organizing health data and subsequently manipulating a digital record by recording and retrieving information. A participant mentioned that they would especially want to use it with a web-based notebook. They also specifically described the usefulness of maintaining documentation in the context of medication management. For example, the participants expressed that a VA could support the recording of drug reactions and the monitoring of medication adherence:

I think keeping notes, like being able to just speak out loud, and if it automatically set a date and a time for when I spoke to it with an observation that was important...if I wanted to record something about the medication.

Participant 10, experienced

I could ask my specific question: “Alexa, did [the patient] take [their] hydromorph contin today?”

Participant 5, minimal experience

Second, each participant population in this study discussed the VA functionalities that would affect care coordination. However, the participants had unique expectations regarding the degree to which VAs could provide coordination support. For example, the participants mentioned design functionalities that included setting reminders for medications, communicating with others, and guiding a caregiver through the steps of a medically related task:

If they got a little notice, that was like, “Hey, it’s time for the medication!” I deﬁnitely think it could really be helpful.

Participant 21, minimal experience

Certainly, managing medications, timing, and if I wanted to be reminded.

Participant 10, experienced

The participants expressed that VAs could specifically assist with care transitions to support communication with others. For example, the participants explained that they could use the VA to leave a PSW a personal message to listen to when they arrived at their house. Some participants (3/22, 14%) also suggested that VAs would help them contact their patients or loved ones, health care professionals, or others on the caregiver team:

Well, communication with the PSWs. If I wasn’t here, let’s say when [my spouse]...I couldn’t leave [them] alone in the latter stages. But in the earlier stages, I thought I could go off to the grocery store and leave [them]. That was up until I came home and found [them] in a delirious state and thought that was a mistake. But if I could, and I wanted to, leave instructions for a PSW...

Participant 10, experienced

The family caregivers of children with special health care needs detailed some of the specific contexts where a VA could support teaching their caregivers—for example, guiding caregivers through the steps involved in administering medication or operating a medical device such as a suction machine. To guide a caregiver through tasks, the participants mentioned that the caregiver could individually set a VA to provide instructions for the procedures (participant 13, minimal experience) or examples of exercises (participant 22, experienced). Although the participants who were family caregivers of children with special health care needs in this study currently create teaching materials to support their home care, they expressed that this interaction method might positively influence the engagement of their hired caregivers with their teaching materials, improving respite care:

Taking somebody through the steps of...“This is that schedule,” “This is the bottle of medication,” “This is what it says,” “These are the steps you go through to safely measure and administer medications.” And it can be generic...“Don’t touch the pills,” “How to put powder in a syringe and then suck water up in it without losing all the powder.”

Participant 2, minimal experience

Family caregivers of older adults also described the use of VAs as a tool to provide instructions to caregivers where the addition of a visual representation for the steps involved in a task may improve the caregivers’ capability to carry out the physical actions:

There might be able to be demonstrations of how to care for certain physical elements...Guide you...But even if it could be done, if there was a screen, if it could be done pictorial.

Participant 10, experienced

Finally, beyond directly supporting a caregiver’s tasks in the home, the participants in this study described the use of their patient or loved one interacting with the VA. They expressed that VAs could support self-care by providing autonomy in managing their medications and supporting cognitive processes and as friendly assistants to interact with during medical procedures. For example, a participant already used the reminder functionality afforded by Google Home to provide their child, who was beginning to take more responsibility for their care, with more autonomy in taking their medications:

We had the medication set up all around, kind of in [their downstairs] apartment. So, we set it up, you know, “set a reminder for [them] to take the pills on top of your white freezer with the Green Cup at 8:00.”

Participant 8, experienced

For adults who may have mild cognitive impairment or physical disabilities, the participants expressed that VAs could support their autonomy through reminders about their care. For example, a caregiver mentioned that VAs could help an older adult through reminders, specifically when to expect their hired care to arrive, without finding the information physically:

If you could have said things like, “Siri, what time does my home care person arrive?” And if it could have given the appointment time to [them] verbally [they] wouldn’t have had to search through papers.

Participant 13, minimal experience

The participants mentioned that caregivers could interact with a VA to check whether a patient or loved one had taken their medication. A care receiver could check their complete medication history using a command. The participants also discussed the importance of supporting cognitive processes to keep older adults oriented with their environment and assist with medication management through verbal cues:

Having a verbal cue for the person to take their medication but as a backup. Seeing if it has been done.

Participant 5, minimal experience

I’m beginning to think...something to remind you when and how often you’ve taken your medication would be good.

Participant 14, minimal experience

In the context of interacting with VAs during a medical procedure, a participant described that a VA could interact with their child to keep them calm while they changed their tracheostomy:

[My child] could like use it to talk [them] through a medical procedure, and that might calm [their] anxiety down a bit...And just like in a kid-friendly way...that would be cool to have in-home.

Participant 1, minimal experience

#### Perceived Ease of Use

In this study, participants with varied experiences using a VA commented explicitly on the ease of using a VA in a home care context, organized into two subthemes: (1) navigating information efficiently and (2) usability concerns. First, the participants mentioned that VAs would ease their documentation. They also expressed that interacting by voice would facilitate recording and retrieving information as it only takes as long as they need to talk. A participant also commented that a voice-based system could instantaneously give information compared with a paper notebook.

The participants described the affordance of multitasking that a VA could provide. They expressed that, while working on a task, they could speak to the system and have health information documented directly during that moment. The ease of recording by voice may reduce the burden of physically writing information on paper; however, the participants still desired to obtain a physical copy of the data if needed:

Sometimes I’m in the middle of doing something else...and I need to remember this thing. But if I stop what I’m doing, then...maybe it’s not that simple to just stop what I’m doing. Or if I wait until the end, I’m going to forget because I just don’t have a very good memory...

Participant 3, experienced

The ease of record keeping by voice could also support a caregiver’s capacity to perform aftercare. For example, a participant mentioned that, if their child were having a seizure, they would be able to physically care for them while maintaining accurate documentation of the event:

If my [child’s] in the middle of a seizure: “Siri, note that [they] had started a seizure at this time,” “Siri, note that [they] stopped,” so I’m not having to wait for [them] to get done and try to remember all the time.

Participant 16, minimal experience

Second, despite the design functionality of VAs that would ease documentation, there were essential concerns regarding this method of interacting with health information. A caregiver (participant 3, experienced) mentioned that using a VA may not be a more straightforward method for managing their child’s health information. However, they first expressed the need to integrate the technology into their routine to determine whether it would be a valuable alternative to other technologies, processes, or practices. There were also concerns about their voice commands being accurately understood by a VA, which may lead to a problematic interaction:

[Siri] just...it wouldn’t register what I was saying...if I have that [for home care], is it going to even register what I’m saying?

Participant 16, minimal experience

A participant was strongly opposed to interacting with VAs in complex home care. Their perceived trust in VA technology, hesitations about information privacy, and the accuracy of recording information by voice negatively influenced their perceived ease of use. Although the participants identified the need for all members of the caregiver team to be comfortable interacting with the VA, conflicting beliefs about the ease of record keeping using a VA might negatively influence care coordination:

I don’t think it’s a good idea; I don’t like that idea. Things can get messed up. You know, certain things could be left out. I mean, it’s always glitches with computers, and they frustrate me all the time.

Participant 4, experienced

Another participant mentioned an essential caveat for technology such as VAs being easy to use. Although they believed that VAs might support individuals with mild cognitive impairment, their concern was that this might negatively influence their physical activity as other technologies have done in the past:

I must admit I have real reservations about them; the more electronics do for us physically...The two things that, for health for seniors and keeping them in their home, they have to have mobility, and I mean I can see it supporting cognition. Things to keep them in their home longer. It’s like the remote on the TV. That getting up and moving to turn on the TV used to be sometimes the only activity those seniors see. So, I’m not sure it’s necessarily a good thing in that respect.

Participant 13, minimal experience

#### Attitudes Toward Use

The participants in this study were excited to think about what they could do with the technology. Despite their varied experiences, they initially expressed excitement about integrating digital home care solutions and their willingness to learn a new technology that could support their caregiving tasks (4/22, 18%). The external factors influencing the participants’ behavioral intention to use VAs in complex home care were organized into four subthemes: (1) excitement, (2) implementation concerns, (3) cost, and (4) prior experience. It is important to note that, although the participants did not explicitly comment on how their previous experience with VAs influenced their attitudes, the fourth subtheme was developed and explored through a deeper analysis of the Likert-scale results in the subsequent section.

Concerns about using VAs were grounded in the current methods the participants used to document health information in the home. As a first example, a participant (participant 4, experienced) explained that health information should not be obtained from a VA but should come directly from the patient or other caregivers. Another participant mentioned that using VAs for medication management may not be as accurate as their current system for tracking their child’s complex medication regimen, which currently provided a physical cue for measuring adherence:

For example, remembering to take [their] meds. I don’t know that I would use [a voice assistant] for that, and the reason being...you can forget to tell it that [you] took it, but...my little pillbox doesn’t lie. So, if it’s in there, I know you didn’t take it. [There’s] no “I just forgot to tell it,” “I actually did take it,” kind of thing.

Participant 3, experienced

Privacy of information was also an essential concern for the participants. In one situation, the family members of a participant (participant 12, minimal experience) influenced them not to purchase a VA based on the perception that they will always listen to what is going on in their homes. Another participant (participant 4, experienced) further expressed concerns about others accessing someone’s health information stored on VAs.

Finally, although the financial cost was an initial concern mentioned by a participant in this study, a hired caregiver also noted the cost of their current documentation methods to our environment and how the use of VAs could support the reduction of that cost:

When it’s paper-based, it’s basically really a big waste...of paper. So, at least if you’re just using Alexa or a voice assistant...it would be at least...let’s say...kinder to nature...If we’re looking at [my client’s] records of [their] things, whenever we try to record the chart, we basically have a load thick of this paper.

Participant 20, experienced

### Likert-Scale Results

#### Overview

**Figure 2:**
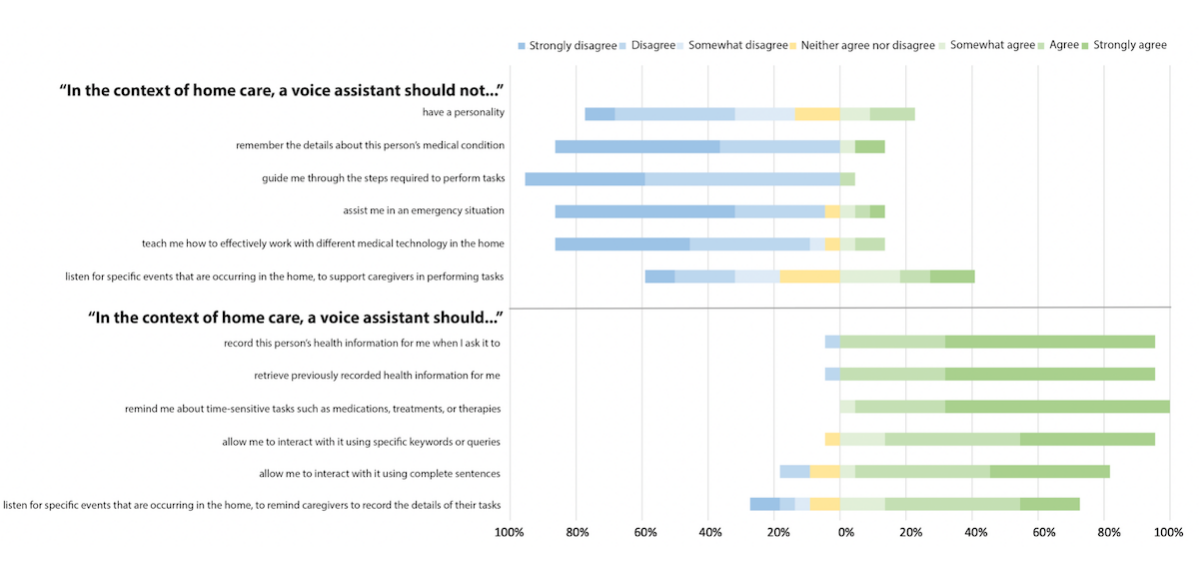
Caregiver expectations of voice assistants in complex home care (N=22).

The caregivers’ initial mental models on using VAs were analyzed to provide insights into how design decisions may affect the successful integration of VAs into complex home care. The Likert-scale questions were used after the interview to prompt additional discussion on the potential features of a VA for complex home care. The Likert-scale questions captured the participants’ initial perspectives on specific design features for VAs in complex home care while exploring their opinions on potential functionality beyond their current mental models. We represented these results graphically to visualize aspects of VA expectations (Figure 2).

Overall, most participants agreed that a VA should record someone’s health information when they request it (21/22, 95%) and retrieve previously documented information (21/22, 95%). They also agreed that a VA should remember the details of someone’s medical condition (19/22, 86%), with the requirement that the data not be stored in a publicly accessible database:

As long as there’s privacy, I think it should. It should be able to retain it. If I came in as a home care nurse or PSW, even as a family member, and I say, “When did this happen?” I don’t have to go back through my notes. My machine can testify who did the treatment last. I mean, that would be very helpful...Anything that records, and I don’t have to chart, I’m on board!

Participant 13, minimal experience

All the study participants (22/22, 100%) agreed that VAs should remind them about time-sensitive tasks such as medications, treatments, or therapies. With respect to interaction preferences with VAs, the participants often expressed their desire to have the option to speak using specific keywords (21/22, 95%) and complete sentences (18/22, 82%).

**Figure 3:**
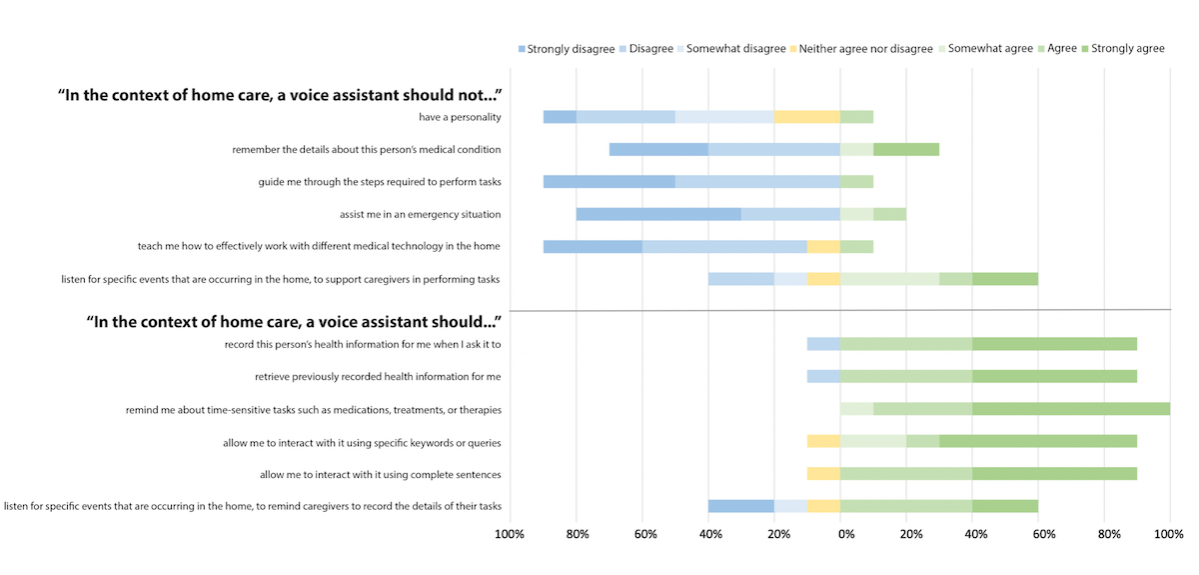
Caregiver expectations of voice assistants in complex home care—experienced (n=10).

**Figure 4:**
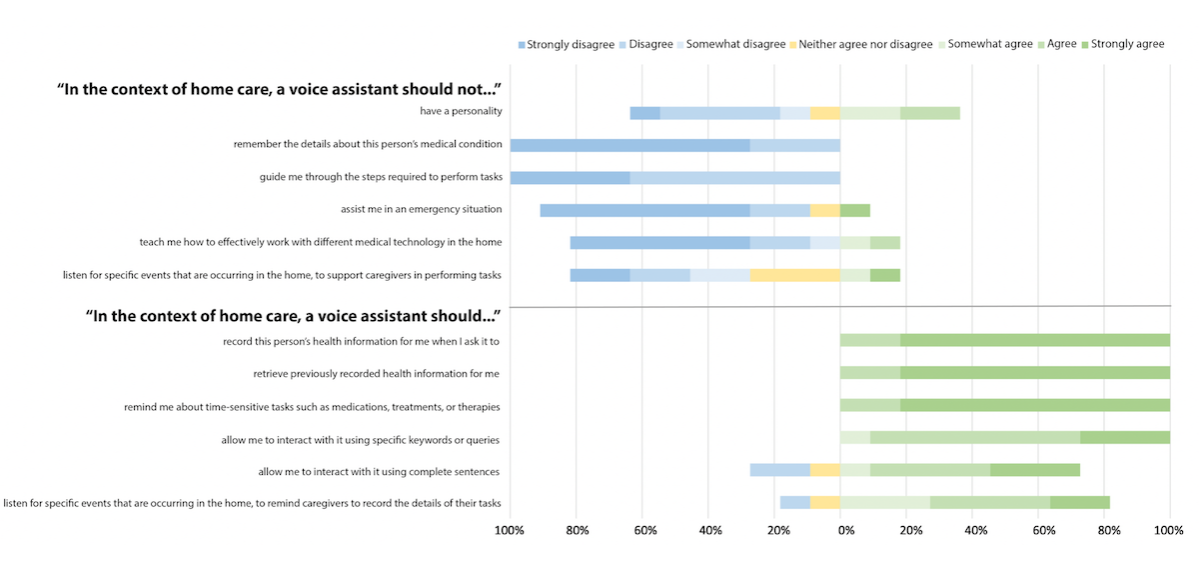
Caregiver expectations of voice assistants in complex home care—minimal experience (n=11).

For more dynamic interactions, most participants agreed that VAs should guide them through the steps required to perform tasks (21/22, 95%), teach them how to use different medical technologies in their homes (18/22, 82%), and support them in an emergency (18/22, 82%). However, they were relatively less agreeable about VAs having a personality (14/22, 64%). The participants were also less agreeable about VAs listening for a particular activity in the home to remind caregivers to record the details of their tasks (16/22, 73%), where more experienced participants (3/10, 30%) disagreed that VAs should listen in this context compared with minimally experienced participants (1/11, 9%). The remaining participants (2/22, 9%) were unsure. Fewer participants (9/22, 41%) felt that a VA should listen for a specific activity in the home to support caregivers in performing their tasks, whereas more experienced participants (6/10, 60%) agreed that it should not compared with minimally experienced participants (2/11, 18%). The remaining participants (4/22, 18%) were unsure. The contrasting perspectives on VA personality, support in an emergency, teaching or guiding caregivers, and listening to activity in the home are further visualized in Figures 3 and 4 and described in the following sections.

#### VAs With a Personality

VA personality may be attributed to cognitive, emotional, and social human characteristics [45]. However, the participants in this study were not provided with an official definition when responding to the personality-based Likert-scale question, which may have influenced the degree of contrasting perspectives that was observed. Regardless, the participants often qualified their responses, providing clarification on their expectations of this quantitative measure.

The participants who agreed that a VA should have a personality expressed that they would want it to be happy and positive. A participant qualified that VAs provide objective responses; therefore, it would be acceptable for a VA to express a personality. Although most participants agreed that VAs should have a personality, more participants who had minimal experience with VAs agreed that a VA should not have a personality (4/11, 36%) compared with participants who had expressed having more experience using VAs (1/10, 10%). The remaining participants (3/22, 14%) were unsure about this potential feature.

The participants who disagreed about VAs having a personality were particularly concerned about the influence that a personality from a device could have on vulnerable caregivers in specific contexts. For example, a *happy* demeanor in a VA providing information about missing scheduled medications could inappropriately influence caregivers’ attitudes toward medication adherence. A participant argued that the vulnerability of caregivers should therefore play a role in designing a VA personality for home care:

I strongly feel that it shouldn’t have a personality...I think that could take advantage of vulnerable people...I know that there’s an argument to be made the exact opposite—that it would make it more user-friendly, it would make it warmer, it could be a companion to the person, etc. There’s a lot of lines you can cross...

Participant 7, minimal experience

They observed the benefit of a VA personality being potentially more user-friendly and acting as a caregiver itself. However, the concept of a VA displaying emotion may inadvertently manipulate the caregivers’ perception of care, which could be particularly harmful in medically fragile situations.

#### Assisting in an Emergency

Most participants (18/22, 82%) agreed that VAs should assist caregivers in an emergency, and this outcome was relatively balanced between experienced and minimally experienced participants (Figures 3 and 4). However, the participants who disagreed about this potential VA feature expressed that the ability of a VA to assist in an emergency should be limited to calling emergency services (eg, calling 911). Calling for the help of another human should be the extent of a VA’s support in this type of situation:

[With] the medical conditions my [child] has...I don’t think I’m anywhere near trusting a device...Yeah, not yet.

Participant 9, experienced

A home care situation may be too complicated for a VA to provide help if there is an emergency. There are likely several factors of the environment and the situation with the child or older adult that the VA cannot perceive. Trust was a concern for some participants (2/22, 9%) in this context, where the VA would need to be 100% accurate in its response if they were to trust it completely.

#### Teaching and Guiding Caregivers Through Tasks

Guiding caregivers through tasks in the home was a potential VA functionality that many caregivers (21/22, 95%) mentioned should be supported. However, other participants (4/22, 18%) also noted that VAs should not be *initially* teaching caregivers how to perform tasks that they have never done before:

Some of my hesitation was that I was defaulting to the importance of face-to-face. If you’re training a new nurse, from my experience, you want someone there on the premises training you in-person: One, for the registered staff to have confidence in the new person, new trainees’ ability, but also, I would think to instill more confidence in the patient in the new caregiver.

Participant 5, minimal experience

The participants emphasized the importance of having in-person training and the need to set access limitations for specific caregiver populations, especially in learning how to use a device that dispenses medication. There was also a concern about a VA providing information about accessing medication-dispensing equipment that could endanger patient safety.

#### Listening to Events in the Home

An always-on VA capable of unprompted responses was seen either as a privacy issue or as significant support for home care safety. Concerning privacy, the participants expressed that they did not like the idea of VAs being present and having the ability to speak without previous notice. Although the participants mentioned that they observed the VA’s potential to notify them about safety events concerning the care situation, other participants said that they would not be comfortable with unprompted interactions. If the VA could respond without being prompted, the participants expressed that this would be an invasion of the private activities in their homes:

In some situations, that could be of significant support and...some situations, that might also be like an invasion of privacy.

Participant 2, minimal experience

The participants expressed that unprompted responses from the VAs would support peace of mind for their respite care concerning safety. A participant described that unprompted responses from a VA could be used to remind their PSW where to stand when performing physical therapy with their spouse:

That would be great for me because I’m not in the room when these caregivers come, and they’re going to be the ones to tell them to stand behind [my spouse].

Participant 17, minimal experience

Finally, a participant mentioned that VAs could listen for unexpected accidents in the home, such as a fall, and promptly notify caregivers to act on issues. They also noted the potential for VAs to identify caregiver abuse:

That could be a huge safety component...to identify caregiver abuse...because really there is caregiver abuse...

Participant 13, minimal experience

## Discussion

### Principal Findings

This study captured the initial perspectives of a sample of caregivers regarding the acceptance of VAs to inform digital technology design for complex home care. This study identified the importance of utility and ease of interaction in influencing technology adoption. The expectations for VAs to support caregivers in managing and communicating health information may positively affect caregivers’ desire to integrate VAs into complex home care while being influenced by previous experiences using VAs. Triangulation of qualifying responses with the Likert-scale results also identified critical design concerns and ethical considerations for using VAs to support caregiving. In the following sections, we discuss the importance of designing VAs for usefulness, ease of use, and the context within which a VA may be used in complex home care.

### Designing for Usefulness

**Figure 5:**
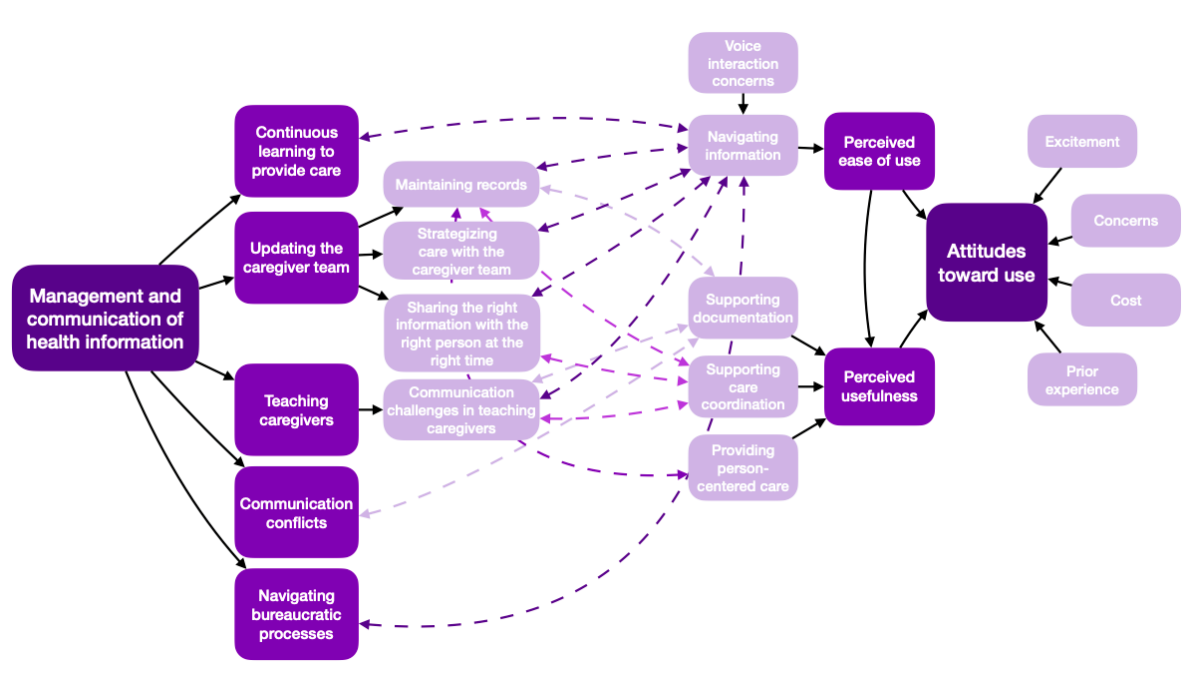
Mapping information management and communication processes with expectations of voice assistants in complex home care.

Previous research on complex home care has formatively identified some of the high-level health information management and communication processes of caregivers in the context of children with special health care needs [26]. With these previous findings and the outcomes of this study on caregivers’ initial beliefs about the design functionalities that VAs could provide in complex home care, we can begin to map the design of VA technology to the home care work domain. Several connections can be made between caregiving tasks and caregivers’ perspectives regarding the ease of use and usefulness of VAs for complex home care that may ultimately influence their attitudes toward integrating this technology (Figure 5).

The utility of VAs is one of the primary motivators for individuals to use this technology in their home [46]. However, understanding the specific factors influencing their utility requires a deeper understanding to inform practical guidelines for developers [46]. Our study can begin to inform the factors influencing VA utility for its use in complex home care. Caregiver participants perceive the utility of VAs in the context of documentation, care coordination, and provision of person-centered care.

Similar to the findings of Sezgin et al [7], this study identified that both family caregiver and hired caregiver participants felt positively toward VAs in the context of recording health information by voice. Family caregiver participants particularly expected VAs to improve the organization of records that could be quickly updated and retrieved in their homes. The utility provided through organized, accessible information could reduce the burden on caregivers to communicate information to others. It may also alleviate conflicts from miscommunication with other caregivers when caregiving teams are large. Furthermore, rather than relying on the primary caregiver to ensure that documented updates are communicated to incoming caregivers, the VA could be used to communicate health information summaries, trends, and other necessary documentation to caregivers. This functionality could relieve the primary caregiver from having to report these details repeatedly.

Ensuring that complete and accurate information is shared about home care can be a challenging task when there are multiple caregivers involved with various responsibilities [25]. An important task that VAs could support is shift handoffs for hired caregivers. A VA for home care built into a mobile app or smart speaker could capitalize on location and scheduling information to provide context-specific details about previous care activities that occurred in the home, supporting a caregiver’s situational awareness before the beginning of their shift. Verbal reminders from a VA located in the house about time-sensitive tasks could further support caregivers’ memory to perform specific tasks or track information when providing care to multiple individuals in a single day.

The participants in this study particularly observed the utility of VAs interacting with the care receiver. Although this was not the focus of this study, the participants identified the impact that VAs could have on supporting self-management of care in the home for children with special health care needs and older adults. The proposed use of VAs by these populations has been previously identified by Sezgin et al [12,13] for applications such as medication tracking under parental, guardian, or caregiver supervision and other health documentation tasks [7]. Research has been directly conducted on children’s interactions with VAs, providing evidence for their positive interactions with this technology in general contexts [47-50]. The participants in this study explained that a VA could provide emotional support to children with special health care needs during potentially uncomfortable medical procedures or for medication adherence, supporting caregiving beyond aspects of health information management and communication. Future research should explore the use of VAs to help children and older adults in these contexts.

### Designing for Ease of Use

In complex home care, the exchange of information is fundamental to the successful outcomes of learning, sharing knowledge, teaching, communicating, and the bureaucratic processing of caregiving [51-53]. Information is dynamically flowing among caregivers within these subdomains of the work environment. However, the information structure in paper-based systems or mobile apps can make accessing it challenging [26]. One of the primary benefits that VAs provide compared with graphical or other physical user interfaces is the removal of visual hierarchies by accessing information through voice commands [13,34].

However, the findings from this study suggest that there is an influencing factor of ease-of-use concerns that may negatively affect information navigation through voice controls. If the VA cannot understand a user’s speech, the ease of use will be severely affected in relation to being error prone. VA technology that supports individuals with speech impairments, such as Google’s *Project Euphonia*, is a critical research area that should be appropriately addressed to successfully integrate this technology into complex home care [54].

### Designing for Use Context: VA Personality

With their inherent communication mode being conversational and potentially human-like—attributing it to cognitive, emotional, and social human characteristics [45]—it is crucial to consider the potential influence of VA personality on the use of this technology [46]. Baptista et al [55] previously identified that personality could influence the users’ perceptions of a VA’s role in health care. In their study, participants perceived the personality of an embodied VA for diabetes management as a friendly coach more than a health professional [55]. A scoping review by Car et al [56] identified other personality traits in studies with VAs in a health care context: informal, human-like, culture-specific, factual, gender-specific, and conversational agent. Given the exploratory nature of our study, the participants were not provided with an initial definition of VA personality or examples of what the personality of a home care VA could be when answering this Likert-scale question, which may have influenced the differences in their responses.

As a result of this nongrounded approach, the use context was identified as a critical factor in caregiver participants’ expectations of a home care VA expressing potential cognitive, emotional, or social characteristics. When designing VA personalities for family caregivers, this population can be considered vulnerable; it is essential to consider the influence of personality traits on their reliance on this type of technology in different caregiving situations. Although the design of VAs currently includes human-like personality traits for health care applications in specific contexts, such as adherence to active living regimens and psychological difficulties [57,58], the participants in this study expected VAs to assist in more than one context. A consistent personality trait for VAs may not be appropriate for every home care situation and may negatively influence a caregiver’s perception. Future research should explore how personality traits influence caregiver engagement, reliance on technology, and medical decision-making.

### Designing for Use Context: Intelligent Support

The extent to which the participants in this study initially expected a VA to assist their tasks suggests that caregivers might prefer a less intelligent VA that is limited to providing a means for retrieving previously entered information. Insights into or interpretations of health information may be an unexpected output from a VA by caregivers while also posing a risk of adverse events [13]. The caregiver participants in this study mainly wished to direct the interaction with VA technology, where the information exchange was not expected to advance beyond their initial intents. Intelligent VAs may be better integrated as complementary caregiving tools [59]. For example, our participants discussed using a VA to create reminders or instructions for procedures based on the information they would consciously provide to the system. When they need assistance, they would prefer to contact other caregivers through the VA rather than asking the VA itself to assist them despite its potential knowledge base.

Finally, although context-specific interactions may improve engagement and adoption of VAs by general consumers, this functionality may require predictive algorithms based on enormous amounts of data about the home to support the system’s intelligence [60,61]. With the uniqueness of the participants’ caregiving backgrounds and home care experiences, some participants would be positively inclined toward a VA that provides context-specific support through passive information collection. However, collecting audio data about the home environment raises ethical considerations. It is essential to consider how these data are used to report home events ranging from accidents to potential caregiver abuse, especially for user groups who find it challenging to navigate the complexities of security choices for Internet-of-Things devices [62]. Caregivers may be concerned if there is no option to control the always listening and analyzing functionality [63].

### Strengths and Limitations

The nature of this exploratory study on the participants’ initial expectations of using VAs in complex home care captured the unique perspectives of the potential primary users of this technology. There has been no significant research conducted using the TAM for understanding VA acceptance and none captured during COVID-19. Although demographics are limited, they offer preliminary insights into diverse situations.

Future work should expand on these results to examine more viewpoints, including people being cared for, various health care professionals, regulators, and technology experts, ultimately bringing a holistic understanding of the system itself and its potential. Additional studies should also examine the potential of VA personality with respect to specific cognitive, emotional, and social human-like attributes and its impact on caregiver perceptions of care, as well as the potential of other methods of conversational interaction with digital tools such as text-based or visual interfaces. An increased sample size through further research would provide more insight into differing caregivers’ perspectives on VAs in complex home care.

### Conclusions

This study provides early emerging research into understanding caregiver perspectives on VAs to support complex home care using the TAM supplemented by a Likert-scale questionnaire. The results point toward the factors influencing the utility of VAs in this work domain and how the ease of interacting with health information through a VA may influence technology adoption. VAs could provide utility for caregivers’ current health care documentation methods and care coordination in the home. There is a desire for VAs to support care recipient independence in the contexts of children with special health care needs and older adults beyond the aspects of information management, providing opportunities for further studies.

Beyond health information interaction, there are ethical considerations for using a VA that provides contextually specific insights from collected audio data given the complexity and diversity of activities occurring in the home. The design of a VA personality should carefully evaluate its potential influence on vulnerable caregiver populations’ perceptions of care. Future research should focus on integrating VAs into specific contexts of information management and communication for complex home care to further understand the factors influencing utility, ease of use, and adoption in the design of this technology.

## Abbreviations

PSW: personal support worker
TAM: technology acceptance model
VA: voice assistant
